# Targeting miRNA by CRISPR/Cas in cancer: advantages and challenges

**DOI:** 10.1186/s40779-023-00468-6

**Published:** 2023-07-17

**Authors:** Bashdar Mahmud Hussen, Mohammed Fatih Rasul, Snur Rasool Abdullah, Hazha Jamal Hidayat, Goran Sedeeq Hama Faraj, Fattma Abodi Ali, Abbas Salihi, Aria Baniahmad, Soudeh Ghafouri-Fard, Milladur Rahman, Mark C. Glassy, Wojciech Branicki, Mohammad Taheri

**Affiliations:** 1 Department of Biomedical Sciences, Cihan University-Erbil, Erbil, Kurdistan Region 44001 Iraq; 2 Department of Clinical Analysis, College of Pharmacy, Hawler Medical University, Erbil, Kurdistan Region 44001 Iraq; 3 Department of Pharmaceutical Basic Science, Faculty of Pharmacy, Tishk International University, Erbil, Kurdistan Region 44001 Iraq; 4 Medical Laboratory Science, Lebanese French University, Erbil, Kurdistan Region 44001 Iraq; 5 Department of Biology, College of Education, Salahaddin University-Erbil, Erbil, Kurdistan Region 44001 Iraq; 6 Department of Medical Laboratory Science, Komar University of Science and Technology, Sulaymaniyah, 46001 Iraq; 7 Department of Medical Microbiology, College of Health Sciences, Hawler Medical University, Erbil, Kurdistan Region 44001 Iraq; 8 Department of Biology, College of Science, Salahaddin University-Erbil, Erbil, Kurdistan Region 44001 Iraq; 9 Center of Research and Strategic Studies, Lebanese French University, Erbil, 44001 Iraq; 10 Institute of Human Genetics, Jena University Hospital, 07747 Jena, Germany; 11 Department of Medical Genetics, School of Medicine, Shahid Beheshti University of Medical Sciences, Tehran, 374-37515 Iran; 12 Department of Clinical Sciences, Malmö, Section for Surgery, Lund University, 22100 Malmö, Sweden; 13 Translational Neuro-Oncology Laboratory, San Diego (UCSD) Moores Cancer Center, University of California, San Diego, CA 94720 USA; 14 Faculty of Biology, Institute of Zoology and Biomedical Research, Jagiellonian University, 31-007 Kraków, Poland; 15 Urology and Nephrology Research Center, Shahid Beheshti University of Medical Sciences, Tehran, 374-37515 Iran

**Keywords:** CRISPR, CRISPR/Cas9, CRISPR/Cas12, Gene editing, miRNAs, Cancer therapy

## Abstract

Clustered regulatory interspaced short palindromic repeats (CRISPR) has changed biomedical research and provided entirely new models to analyze every aspect of biomedical sciences during the last decade. In the study of cancer, the CRISPR/CRISPR-associated protein (Cas) system opens new avenues into issues that were once unknown in our knowledge of the noncoding genome, tumor heterogeneity, and precision medicines. CRISPR/Cas-based gene-editing technology now allows for the precise and permanent targeting of mutations and provides an opportunity to target small non-coding RNAs such as microRNAs (miRNAs). However, the development of effective and safe cancer gene editing therapy is highly dependent on proper design to be innocuous to normal cells and prevent introducing other abnormalities. This study aims to highlight the cutting-edge approaches in cancer-gene editing therapy based on the CRISPR/Cas technology to target miRNAs in cancer therapy. Furthermore, we highlight the potential challenges in CRISPR/Cas-mediated miRNA gene editing and offer advanced strategies to overcome them.

## Background

In 1987, the first instance of clustered regulatory interspaced short palindromic repeats (CRISPR) was found in the bacteria *Escherichia coli* K12 [1]. For the past 20 years, these palindromic repeats have been discovered in approximately 40% of bacteria and 90% of archaea [2]. CRISPR has repeat sequences that are spaced by exogenous nucleotides from plasmids or viruses that have invaded, and its loci are frequently surrounded by some related endonucleases, like CRISPR-associated protein (Cas). First, precursor CRISPR RNAs (pre-crRNAs) are produced from CRISPR. The resulting crRNAs bind to the Cas protein to form a complex that can activate the transcription of certain DNA regions [3]. Although the main activity of this ribonucleoprotein (RNP) complex is to cleave specific DNA locus, specified by the crRNA sequence, with the nuclease activity of the Cas protein. There are three stages to the immune response in all known CRISPR/Cas systems: 1) CRISPR arrays can undergo adaptation and spacer acquisition, in which a fragment of the invading genome is added to the existing gene, 2) mature crRNAs [guide RNA (gRNAs)] are expressed as a result of the CRISPR array processing, and 3) interference, wherein the gRNAs direct Cas proteins to the target location of the invaded genome for destruction or cleavage [4, 5].

There are various classes and types of CRISPR systems, the widest one is class 2, CRISPR/Cas9. Here, in CRISPR/Cas9, the Cas protein works in conjunction with a chimeric single-guide RNA (sgRNA) made from crRNA and tracrRNA. TracrRNA is necessary for Cas nuclease activity, while crRNA detects and binds sequences next to the protospacer adjacent motif (PAM), 5′-NGG-3′, on the target DNA sequences [6]. The target DNA sequence is complementary to the first 20 nucleotides of the sgRNA, which are then followed by a sequence known as PAM, which is generally NGG [7].

The CRISPR/Cas system has potential applications in medicine, including diagnostics, therapeutics, and drug screening. Despite the growing popularity of the CRISPR/Cas technology for gene editing, being used in studying microRNAs (miRNAs) remains mostly undefined [8].

In addition, studies revealed that applying CRISPR/Cas is significantly less expensive, has a lower chance of contamination, and is more accruable and specific in its ability to target miRNAs in cancer therapy, when compared with the current miRNA studying approaches [9].

Small non-coding RNAs known as miRNAs influence gene expression by acting as either transcriptional regulators or translational repressors of their downstream target genes [10]. In mammals, it is expected that almost half of all protein-coding genes’ activity is regulated by miRNAs, which are highly conserved non-coding regulatory factors [11]. In human malignancies, miRNA expression is dysregulated by a number of processes, including miRNA gene amplification or deletion, improper miRNA transcriptional regulation, dysregulated epigenetic alterations, and errors in the miRNA biogenesis machinery [12–16]. Furthermore, miRNAs dysregulations have been demonstrated to influence the characteristics of cancer, such as maintaining proliferative signaling, avoiding growth suppressors, apoptosis resistance [17–19], inducing invasion and metastasis [20], drug resistance [21], and inducing angiogenesis [22]. Therefore, there is a lot of potential for using miRNAs as diagnostic and therapeutic targets in cancer therapy. This study explores the novel insights that have been achieved due to the development of CRISPR/Cas systems as a strategy to target miRNAs in cancer therapy. Besides, we discussed the potential challenges and advanced strategies that can be applied to overcome these challenges.

## Biogenesis of miRNAs and regulatory mechanisms and their role in cancer

**Fig. 1:**
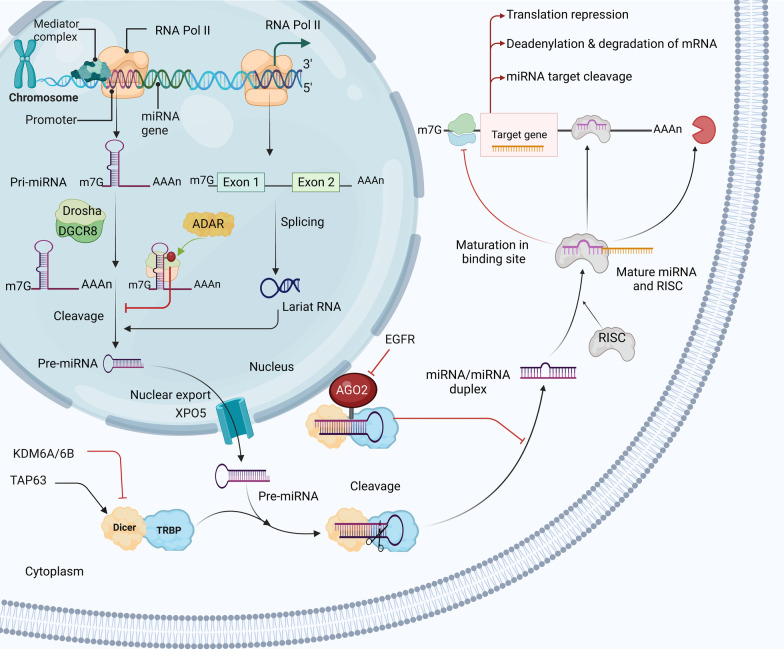
A graphical illustration of miRNA biogenesis. The pre-miRNAs have one or more incomplete hairpin structures that have a stem of about 33 base pairs. Ribonucleases Drosha and Dicer process the pri-miRNA precursor in two separate processes. In the nucleus, Drosha first cuts the pri-miRNA into a pre-miRNA about 70 nucleotides in length, which is then transferred to the cytoplasm by XPO5. The mature, functional, ds miRNA is then processed by Dicer using the pre-miRNA as a template. After maturation, the miRNA is covalently linked to RISC, a multiprotein complex that contains the AGO protein and is essential for RNA silencing. Exon 1 and exon 2 are connected together when the RNA splicing process takes place and leads to the formation of the lariat RNA (circular molecules with a short tail). Following RNA splicing and additional processing, the intron-containing spliced lariat may function as a pri-miRNA for intronic miRNA synthesis. XPO5 exportin-5, ds double-stranded, RISC RNA-induced silencing complex, AGO argonaute, ADAR adenosine deaminase RNA specific, TRBP tar RNA-binding protein, EGFR epidermal growth factor receptor

Single-stranded, non-coding RNAs called miRNAs are derived from primary miRNA (pri-miRNA), an early transcript produced by RNA polymerase II (Pol II) [23]. Approximately 50% of the known miRNAs are made from the introns and a few exons of protein-coding genes. The other 50%, intergenic, are made from their promoters and do not depend on host genes for transcription and expression [24, 25]. Pri-miRNAs are synthesized using the same transcription steps, capping, 3′ polyadenylation, and splicing that are used to make mRNA. DNA-dependent RNA Pol II is the enzyme responsible for transcribing miRNA genes (Fig. 1). The miRNAs can sometimes be transcribed as a cluster, which is a single long transcript. Clusters can have similar or the same seed regions, which means they are a family [26, 27]. Following transcription, the nucleus produces pri-miRNAs with a characteristic stem-loop structure. The miRNA/miRNA duplex is then released after pri-miRNAs undergo two constitutive cuts, which result in pre-miRNAs. Drosha cuts pri-miRNA into pre-miRNA in the nucleus, which exportin-5 transfers to the cytoplasm [28]. Dicer uses pre-miRNA as a template to make mature, functional double-stranded (ds) miRNA [29]. After maturation, miRNA usually binds to a 3′ UTR and either destroys or suppresses mRNA translation. It has been demonstrated that a single miRNA can regulate the expression of numerous mRNAs, and each mRNA could also be controlled by different miRNAs. Based on experimental findings, in most cases, miRNAs bind to specific sequences at the 3′ UTR of their target mRNAs to trigger translational inhibition and the decapping and deadenylation of the mRNAs they target [30, 31]. It has been discovered that miRNA binding sites can also be located in other mRNA regions, such as the 5′ UTR, coding sequence, and promoter regions [32]. In particular, miRNAs inhibit gene expression when they bind to the 5′ UTR and coding sequences [33]. However, miRNAs that bind to promoter regions enhance the transcription process [34]. Nevertheless, additional study is necessary to comprehend the practical importance of this kind of interaction.

**Fig. 2:**
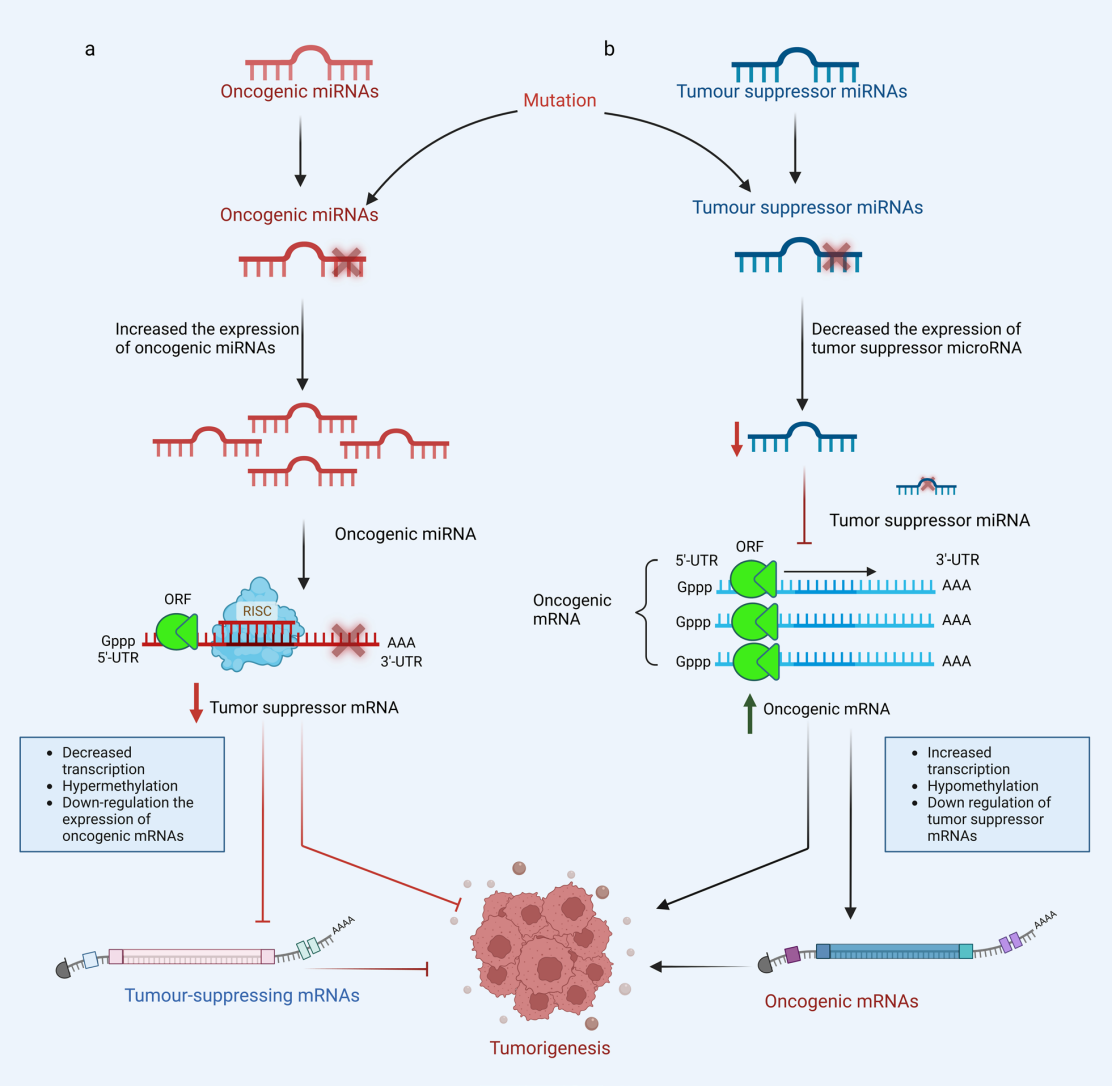
A graphical illustration of how oncogenic and tumor suppressor miRNAs are regulated during tumorigenic events. **a** When oncogenic miRNAs are expressed at higher levels in malignant cells, tumor suppressor gene expression is lowered either as a result of mRNA degradation or hypermethylation. **b** Oncogenic miRNA expression may be increased by decreasing the expression level of tumor suppressor miRNAs. Both oncogenic and tumor suppressor miRNAs contribute to tumorigenesis by promoting a variety of malignant phenotypes, including cell development, anti-apoptotic activity, invading, angiogenic, and spreading. RISC RNA-induced silencing complex, ORF open reading frame

Furthermore, miRNA plays a crucial role in promoting or inhibiting cancer progression through oncogenic or tumor suppressor miRNAs (Fig. 2). Indeed, normal tissues and tumor tissues have different expressions of some types of miRNAs. Thus, from a therapeutic perspective, targeting miRNA has been considered an effective approach in cancer therapy, especially by designing specific miRNA inhibitors to target oncogenic miRNAs that are overexpressed in tumour cells [35]. Pathogenic miRNA alterations can be regulated with miRNA mimics or antagomiRs (anti-miRs), leading to adjusting the gene regulatory network and normalizing the signaling pathways in cancer cells [36].

When miRNA binds to a 3′ UTR, it either destroys the mRNA or inhibits its translation. The degree of miRNA complementarity to the 3′ UTR determines the level of mRNA degradation or translational repression [37].

MiRNAs are a type of non-coding RNA that influence gene expression after transcription has already taken place by interacting with the 3′ UTR of mRNA. Friedman et al. [38] analyzed more than 45,000 miRNA target locations in the human 3′ UTR region, and they found that miRNA regulates roughly 60% of human protein-coding genes.

Dysregulation of miRNA expression has been associated with various pathological conditions, including cancer [39–41]. In cancer, miRNAs can act as either tumor suppressors or oncogenes, depending on the target genes they regulate. The role of miRNAs in cancer progression is complex and involves multiple mechanisms. Some miRNAs have crucial roles in cancer progression, including the control of oncogenes and tumor suppressor genes. For instance, miR-21 is an oncogenic miRNA that targets tumor suppressor genes such as *PTEN* and *PDCD4* [42], while miR-34a is a tumor suppressor miRNA that targets oncogenes such as *MYC* and *BCL2* [43]. Further, miRNAs can promote cancer cell proliferation and survival by targeting genes involved in cell cycle regulation, cell death, and DNA damage response. For example, the miR-17-92 cluster, which is up-regulated in many cancers, promotes cancer cell proliferation by targeting the tumor suppressor gene *PTEN* [44]. Moreover, several miRNAs have been shown to regulate epithelial-to-mesenchymal transition (EMT) by targeting genes involved in cell–cell adhesion and cytoskeleton organization. For example, miR-200 family miRNAs inhibit EMT by targeting the transcription factors zinc finger E-box-binding homeobox 1 (ZEB1) and ZEB2 [45]. Likewise, miRNAs have been found to regulate angiogenesis by targeting genes involved in angiogenic signaling pathways. For example, miR-126 inhibits angiogenesis by targeting vascular endothelial growth factor A (VEGF-A) and phosphatidylinositol 3-kinase regulatory subunit 2 (PIK3R2) [46]. Additionally, miRNAs can also regulate immune responses by targeting genes involved in immune signaling pathways. For example, miR-155 promotes inflammation by targeting the negative regulator of nuclear factor-κB (NF-κB), a suppressor of cytokine signaling 1 (SOCS1) [47, 48]. MiRNAs influence alternative splicing and chromatin remodeling [49]. Dysregulated epigenetic changes of miRNA may induce tumors [50].

It has been discovered that miRNAs play an important role in cancers beyond the intracellular level, for example, in extracellular fluids, either as free circulating molecules or enclosed in exosomes. These extracellular molecules play a crucial role in cell signaling, and they are capable of traveling extensive distances to exert their effects on recipient cells, particularly immune cells in the tumor microenvironment [51].

Overall, miRNAs play critical roles in cancer progression by regulating various cellular processes. Understanding the precise mechanisms underlying miRNA dysregulation in cancer is essential for developing effective miRNA-based therapies for cancer.

Therefore, therapeutically, miRNA has been perceived as a useful method in cancer therapy, particularly in the construction of specific miRNA inhibitors to target oncogenic miRNAs that are overexpressed in tumor cells [52]. Thus, correction of miRNA abnormalities in cancer cells using gene editing tools such as CRISPR/Cas, can restore normal function to the cells’ gene regulatory networks and signaling cascades [53].

## Challenges of current miRNA-based cancer therapy

An innovative path for cancer research and treatment would be possible through the identification of novel therapeutic drugs that can specifically inhibit oncogenic miRNAs [54].

**Fig. 3:**
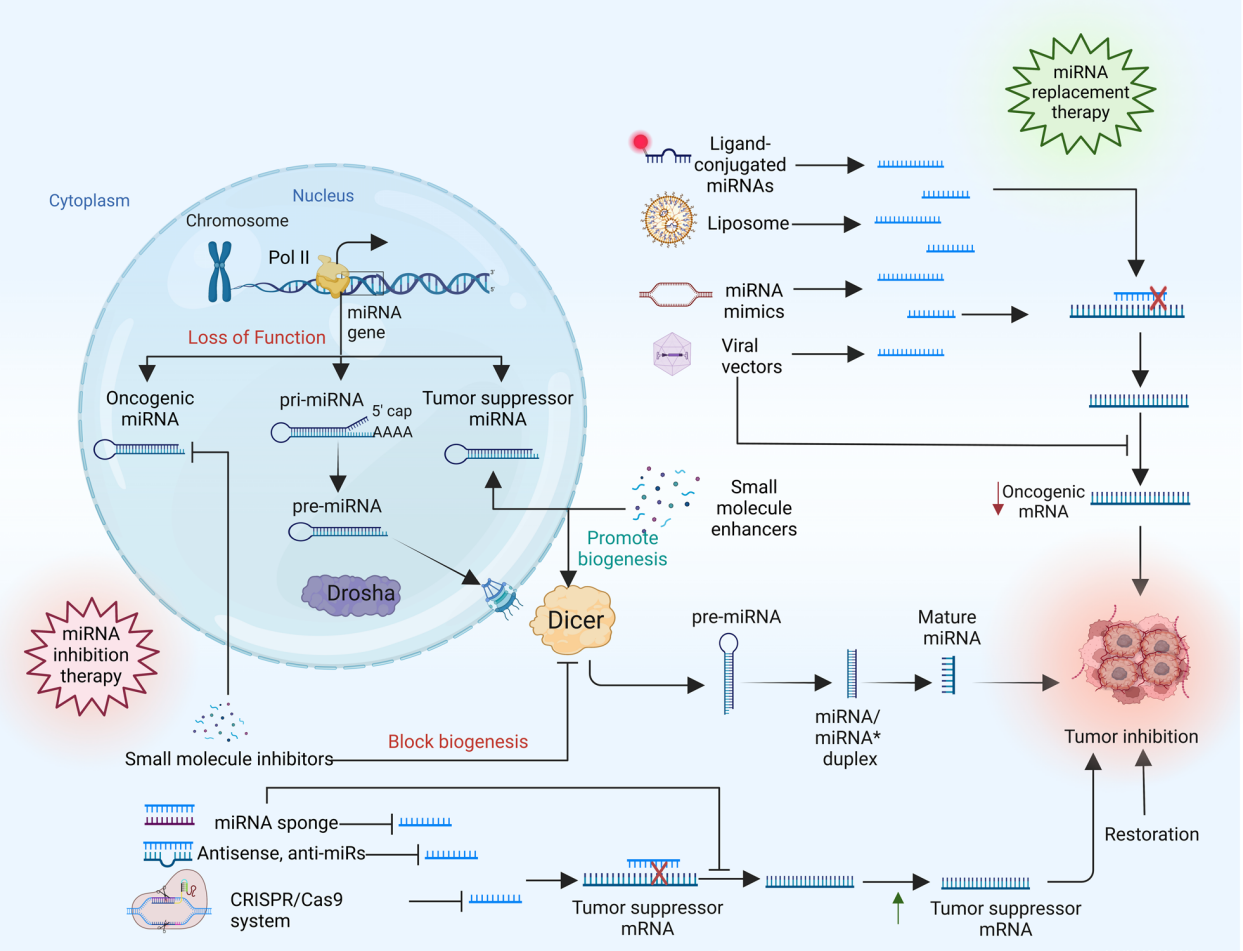
Therapeutic use of miRNAs in cancer treatment. miRNA replacement therapies or oncogenic miRNA inhibition are the two primary current methods utilized to prevent the overexpression and functions of miRNAs. miRNA replacement therapy such as ligand conjugated miRNAs, liposomes, miRNA mimics, and viral vectors are used to suppress the oncogenic miRNAs. Furthermore, miRNA inhibition therapy includes small molecule inhibitors, miRNA sponge, antisense, and CRISPR/Cas9 which inhibit the oncogenic miRNA’s function. miRNAs microRNAs, CRISPR/Cas9 clustered regularly interspaced short palindromic repeats/CRISPR-associated protein 9

Since an imbalance in miRNA expression levels is associated with the development of cancer, miRNA-based cancer therapies are developed with two distinct guiding principles: the suppression of miRNAs that are overexpressed and the restoration of tumor suppressor miRNA function (Fig. 3) [55]. To restore the expression level of tumor-suppressive miRNAs and improve the function of endogenous miRNAs, miRNA mimics and other small compounds are typically used to repair miRNA function. On the other hand, overexpressed miRNAs can be inhibited using small-molecule inhibitors, antagomiRs, and miRNA sponges that have been specifically created to target particular oncogenic miRNAs that are overexpressed in cancer cells [56]. However, each of these techniques, as summarized in Table 1 [57–71], has its limitation, and requires further study to become more effective and less toxic.

**Table 1:** Advantages and disadvantages of current miRNA inhibitors and enhancer

Methods	Type	Description	Examples	Target/function	Advantages	Limitations
miRNA mimics	Enhancer	miRNA mimic is chemically synthesized ds miRNA like RNA, designed to perform similar functions as endogenous miRNA	Let-7 mimic MRX34 (a miRNA-34a mimic)	Delivery of let-7 mimic decreased the prevalence of K-ras-dependent lung cancers [57] miRNA-34a mimics reduced tumor growth in liver cancer [58]	Predicted to target the same sets of genes because they share the same sequence as natural miRNA [59]	Have immune related adverse effects [60] Causes non-specific changes of gene expression due to higher concentration delivery of mimics inside the cells [61] Often chemically modified for stability which might have different effects than endogenous miRNA [61] Might have negative effects because miRNA mimic complimentary strand acts inappropriately [62]
AntagomiRs	Inhibitor	AntagomiR is chemically synthesized single-stranded RNA and specially developed for inhibiting the function of endogenous miRNA	miR-10b antagomir	miR-10b antagomir reduced target protein, homeobox D10, and lung metastasis of breast cancer [63]	Chemical modification gives them higher affinity to cell membrane and higher stability [64]	Found to cause lowering of white blood cells, increase serum levels of bilirubin, ALT and AST [63]
miRNA sponges	Inhibitor	Contains complementary binding sites for target miRNA but produced within the cell by transgene	CDR1as/ciRS-7-lncRNA ZFAS1	Targets miRNA-7 mediated insulin secretion by islet cells [65] Regulates miR-190a-3p to control the development of cervical cancer [66]	Easy to deliver into difficult-to-transfect cell lines or cells in vivo by viral vector [67] Sponges interact with the mature miRNA, thus clustering of miRNA precursors does not affect their effectiveness [67]	Binds in the seeding region of the miRNA, thus might inhibit many genes by targeting many miRNAs of the same family [67]
LNA-modified antisense oligonucleotides	Inhibitor	RNA analogs in which a methylene connection between the 2′-oxygen and the 4′-carbon “locks” the ribose ring	miR-92a-3p	Inhibits miR-92a-3p induced colon cancer cells proliferation [68] Regulates EMT to prevent miR-205-5p-mediated breast cancer metastasis [69]	Chemical modification gives them more affinity to the target and stability towards endogenous nucleases activity [70]	They would increase nuclease resistance and prevent the targeted probe from degradation; they cannot be inserted at the 5′ terminus of the probe or at the nearby nucleotide [71]

*MRX34* liposomal formulation of miR-34a, *ALT* alanine transaminase, *AST* aspartate aminotransferase, *CDR1* cerebellar degeneration-related protein 1, *LNA* locked nucleic acid, *EMT* epithelial-mesenchymal transition

Due to the limited cellular uptake properties used in the current delivery carriers, the primary drawback of miRNA-based therapy is delivery efficiency [72]. Additionally, to accomplish its medicinal objective, it requires the proper delivery of the particles into and via the body’s complex circulatory system as well as through the cell membranes of the various tissues.

The second challenge with miRNA-based therapy is the specificity and off-target effects of miRNA. As previously mentioned, one of the future challenges of miRNA is “multi-targeting” which gives the advance to treat diseases by affecting multiple pathogenesis-correlated targets. However, since the binding between miRNA and target mRNA requires only a partial complementarity, the risk of off-target effects is very high [73].

The third challenge of current miRNA base therapy is the miRNA-induced toxicity, as a result of the off-target occurrence. If miRNAs transcriptionally control the expression of non-target genes, such as drug-metabolizing enzymes, alterations may occur and lead to toxicity. Deregulating the expression of cytochrome P450s (CYPs) and bile acid synthase cholesterol 7 alpha-hydroxylase (CYP7A1) by particular miRNA may change drug metabolism and increase drug accumulation, as in the case of CYPs and bile acid synthase CYP7A1 [74]. The approach of miRNA agomir has shown some significant drawbacks [64]. A human clinical trial demonstrates that the use of MRX34, which is a liposomal mimic of miR-34a, to treat liver cancer in humans has caused clinical toxicity in some individuals. Furthermore, the trial ended as a result of severe immune-mediated adverse impact, which resulted in the loss of four volunteers [75].

The rapid clearance of a naked nucleotide in the bloodstream represents a main obstacle for miRNA-based drugs in vivo and it is considered the fourth main challenge [76]. To evade the degradation by RNase or components of the immune system present in body fluids, the binding and embedding of miRNA molecules with carrier molecules or particles are essential.

On the other hand, CRISPR/Cas system revolutionized miRNA-based cancer therapy by making it possible to quickly alter oncogenic miRNA genes in living cells and animal studies. For instance, CRISPR-based techniques lead to a decrease of several miRNA expressions with high effective rate than traditional methods [77]. Similarly, CRISPR/Cas9 successfully knocked down the expression of oncogenic miRNA in different types of cancer including ovarian cancer cells [78] and brain tumor cells [79]. These findings suggest that CRISPR/Cas can decrease cancer cell survival and multiplication based on miRNA gene knockdown.

Furthermore, CRISPR/Cas gene-targeted cancer therapy is entering preclinical trials [80]. The difficult issue of discovering miRNA targets has been approached in a variety of ways, and it can be modified to target a variety of genes in vivo, underlining its significant promise for future therapeutic use.

## Innovative advances in CRISPR/Cas miRNA-editing technology

As previously reported [81], targeting miRNA for either stimulation or suppression of gene expression had a significant impact on studying cancer biology and as a tool for cancer prognosis and treatment. However, the techniques currently in use still show several limitations and require further studies to improve their safety and effectiveness.

As a result, it has been shown that CRISPR/Cas technology provides a promising new therapeutic approach for miRNA targeting, especially when used as an inhibitor. For example, knocking out miRNA-155 by CRISPR/Cas9 in macrophage cell lines shows a great reduction in the development of rheumatoid arthritis-related symptoms [82]. Moreover, CRISPR/Cas9 shows less stimulation of proinflammatory cytokines when compared to siRNA knockout methods, resulting in higher safety for in vivo application [82, 83].

Several studies have investigated CRISPR/Cas-mediated knockdown of miRNA genes and miRNA transcripts in vivo and in vitro (Table 2 [77, 82, 84–95]). For example, Chang et al. [77] performed CRISPR/Cas9 knockdown on three miRNA genes expressed in two different colorectal cancer cell lines, HCT116 and HT-29. They showed that CRISPR-based techniques lead to a decrease in miRNA-141, miRNA-17, and miRNA-200c expression with an effectiveness rate of 96% higher than traditional control vectors. Similarly, Huo et al. [78] used lentiviral CRISPR/Cas9 constructs to successfully knock down the expression of pre-miR-21 in ovarian cancer cells. This led to the up-regulation of the miR-21 target genes *PDCD4* and *SPRY2*, which inhibited the growth, migration, and invasion of ovarian cancer cells. In addition, El Fatimy et al. [79] demonstrated that down-regulation of miR-10b led to reduced miR-10b levels in glioma and brain tumor cells. These findings provide evidence for the potential of the CRISPR/Cas technology to inhibit the survival and multiplication of cancer cells, as well as down-regulate their expression.

**Table 2:** Targeting miRNA genes and miRNA transcripts by CRISPR/Cas in vivo and in vitro studies

Type of CRISPR	Purpose of use	miRNA	Target	Type of diseases	Cell line (in vitro)	Animal (in vivo)	Vector	Finding	References
CRISPR/dCas9	Therapy	miR-155	–	Liver cancer	HEK293T, AML12	–	Lentivirus	Exosomes are modified to transport and deliver novel RNA payload to target cells	[84]
CRISPR/Cas9	Therapy	miR-155	S PU.1, AID, SHIP1, SMAD5, and SOCS1	RA	RAW264.7 and HEK293T	–	Lentivirus	Genome editing with miRNA-155 holds promise as a treatment for RA	[82]
CRISPR/Cas9	Function investigation	10 miRNA genes	–	–	–	Zebrafish	–	Cas9 nuclease with 2, 4, 10 or > 24 multiplexed sgRNA can cause mutations in 90% of miRNA genome	[85]
CRISPR/Cas9	Therapy and pathway investigation	miRNA-26a-1 miRNA-26a-2	–	Respiratory distress syndrome	–	Mice (C57BL/6J and FVB)	–	miR-26a has a role in PS synthesis in AECIIs	[86]
CRISPR/Cas9	Function investigation	miR-196a miR-219	–	Neurocristopathies	–	Xenopus	–	miRNA mimics have been applied to recover the knockout phenotype Knocking out miR-219 and miRNA-106a related to loss of NC and hatching gland abnormalities in mice	[87]
CRISPR/Cas9	Therapy	miR122	–	HCV	Huh7	–	Adeno-associated virus	Creating tailored cellular clones that resemble to the parental cells yet are immune to HCV multiplication and infection	[88]
CRISPR/Cas9	Function investigation	miR-4018a	–	–	–	Ciona embryo	Electroporation	MICR-ON is used to observe and analyze miRNA expression and function in a living organism and its biological system	[89]
CRISPR/Cas9	Function investigation	miR17 family	Fog2	–	E14	Mouse	–	Varied members of the miRNA17 family (14 miRNAs) have different functions in embryonic stem cell development	[90]
CRISPR/Cas9	Investigation	–	–	–	HEK293T	–	–	A pool of transiently transfected cells must allow functional examination of a hypothesized miRNA-target combination using clonal cell lines or transgenic animals	[91]
CRISPR/Cas9	Therapy	miR-17 miR-200c miR-141	–	Colorectal cancer	HCT116 and HT-29	–	Lenti-CRISPR	Suppressing miRNAs by up to 96% in robustness by using CRISPR/Cas9 CRISPR/Cas9 regulates off-targeting on miRNAs from the same family or with a similar sequence It has been demonstrated how CRISPR/Cas9 miRNA knockdown is stable over the long term	[77, 92]
CRISPR/Cas9	Testing the methods	miRNA-29b1	–	–	–	Mice (C57BL/6)	–	Knocking out miRNA-29b1 gene in mice A 10 bp deletion, a 23 bp loss, and a 3 bp insertion have been seen in mouse genotypes miRNA-29b1 expression was down-regulated in the kidneys, liver, heart, spleen, and lung	[93]
CRISPR/Cas9	Function investigation	miR-31-5p miR-92b-3p miR-146b-5p miR-151a-3p miR-194-5p miR-95-3p miR-181a-5p miR-188-5p miR-196b-5p miR-584-5p miR-1304-3p miR-100-5p miR-149-5p	–	Cervical and gastric cancer	HeLa or NCI-N87	–	Lentivirus	Five HeLa pro-fitness and cervical cancer up-regulated miRNAs were found There was an up-regulation of six NCI-N87 profit and gastric cancer miRNAs Three down-regulated and anti-fitness miRNAs were found	[94]
CRISPR/Cas9	Function investigation	miR-497 miR-195 miR-143 miR-145	–	Cardiovascular diseases and cancer	VSMCs, HEK293T	–	Lentivirus	Editing miR-195 decreased miR-497a expression in the miR-497195 cluster. Despite the absence of gene editing in the miR-497a genomic region, computational simulation demonstrated a change in the three-dimensional form of the pri-miR-497-195	[95]

*RA* rheumatoid arthritis, *AID* acquired immune deficiency, *SHIP1* SH2-containing inositol-5ʹ-phosphatase 1, *SMAD5* SMAD family member 5, *SOCS1* suppressor of cytokine signaling 1, *PS* pulmonary surfactant, *AECIIs* alveolar type II epithelial cells, *NC* neurocristopathies, *HCV* hepatitis C virus, *VSMCs* vascular smooth muscle cells, *bp* base pair, *sgRNA* single-guide RNA, *MICR-ON* miRNA-inducible CRISPR-on system

### CRISPR/Cas-based miRNA gene editing

DNA can be added, removed, or changed at endogenous loci in a genome using the sophisticated genetic engineering technique known as genome editing with sequence-specific nucleases [96]. To produce double-strand breaks (DSBs) on target DNA at a predetermined locus, sequence specific nucleases (SSNs) act as molecular scissors. Endogenous DNA repair processes are triggered in cells by the creation of DSBs. Non-homologous end joining (NHEJ), the predominant DNA repair process in higher eukaryotes, is very error-prone and can result in insertions, deletions, or mismatch alterations at targeted loci. In contrast to NHEJ, homology-directed repair (HDR) needs a DNA donor template and is less efficient [97]. Genome editing has recently become popular due to the development of zinc finger nuclease (ZFN) [98], transcription activator-like effector nucleases (TALEN) [99], CRISPR-Cpf [100], and CRISPR-Cas9 technologies [101].

The first technology to provide a Cas effector for precise genome editing was the DNA-targeting Cas9 system from CRISPR-class 2. Cas9 [102] and Cas12 [103] are two examples of class 2 putative enzymes that have been found as programmable miRNA gene-targeting modules for biotechnological applications due to their widespread use in genome engineering across a variety of species. Both Cas9 and Cas12 use analogous mechanisms to detect and break the complementary DNA sequence that corresponds to the RNA guide [104].

**Fig. 4:**
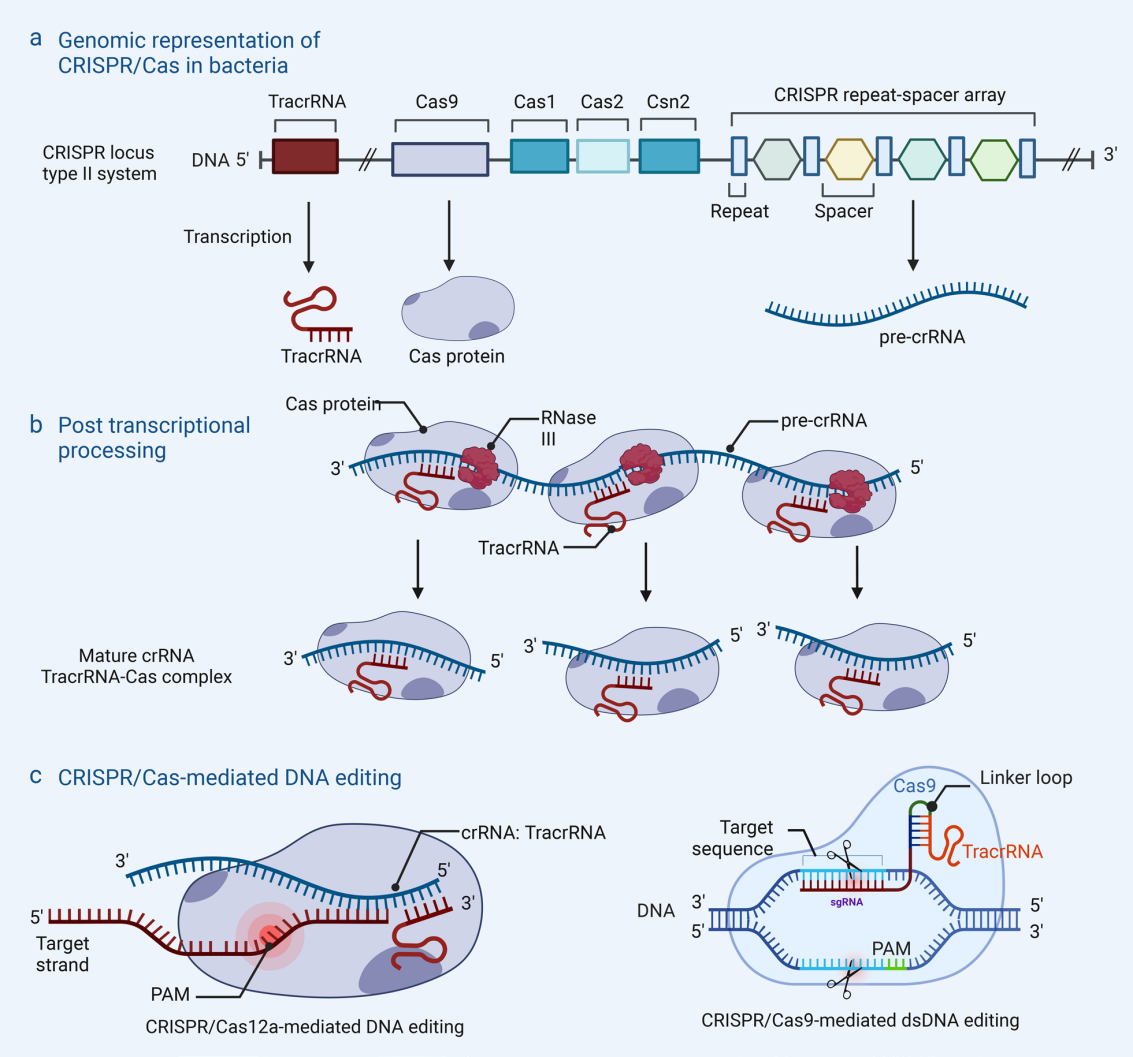
The CRISPR/Cas system and gene editing mechanism. **a** CRISPR/Cas locus in the bacterial genome with associated transcription and translation products. **b** CRISPR/Cas engineered for site-specific gene editing. **c** Editing of dsDNA using CRISPR/Cas12a and CRISPR/Cas9 respectively. CRISPR/Cas clustered regularly interspaced short palindromic repeats/CRISPR-associated protein, dsDNA double strands DNA, crRNA CRISPR RNA, TracrRNA trans-activating CRISPR RNA, sgRNA single-guide RNA, PAM protospacer adjacent motif

Nuclease activity in these RNP effectors is triggered by the identification of a specific sequence of DNA. The RNP or Cas protein scans long DNA sequences and recognizes the PAM region, then, in turn, initiates an ATP-independent DNA unwinding process and finally results in the pairing of the DNA target strand (TS) and the RNA guide [105]. During the RNA–DNA hybridization process, the “non-target” DNA strand becomes unpaired from the targeted strand, and the Mg^2+^-dependent endonuclease exploits two active sites (HNH and RuvC in Cas9) or a single active site (RuvC in Cas12) to cleave DNA strand independently (Fig. 4) [101].

#### Cas9-based mechanism for miRNA gene targeting

Large multidomain CRISPR/Cas9 enzymes of type II vary in size from 700 amino acid residues (subtype II-D) to over 1700 amino acid residues (subtype II-C) [106]. Cas9 is part of a functioning RNP that also includes either a crRNA-tracrRNA scaffold hybrid or an engineered crRNA-tracrRNA fusion sgRNA [107]. Cas9’s bilobed structure, made up of a CRISPR RNA-targeted strand (crRNA-TS) pair recognition lobe (Rec lobe) and a nuclease lobe (Nuc lobe), is responsible for target binding and cutting [108, 109]. Rec1, Rec2, and Rec3 are all subdomains of the Rec lobe, while the Nuc lobe has RuvC, HNH, and wedge-PAM-interacting areas as its subdomains.

For PAM identification, apo-Cas9 must first change from its open state when no gRNA is present to its closed state after guide recruitment [110, 111]. Together with the Rec lobe, the RuvC and HNH nuclease domains change their respective conformations upon target DNA binding to activate the nuclease, resulting in a blunt-ended DNA cut [112].

To find potential sequence targets, Cas9 either uses guided 1D diffusion or random 3D collisions to detect PAM sequences along DNA sequences [113]. DNA treated with N4-cystamine forms disulfide bonds with *Streptococcus pyogenes* that have been modified with cysteine. Modification of Cas9 (T1337C) allowed the temporary interrogation state to be captured and contributed to the understanding of how Cas9 “reads” DNA [114]. Interestingly, by binding to PAM, Cas9 aggressively bends and twists DNA, which causes the nucleotides to flip out of the duplex and toward the gRNA, allowing for interrogation miRNA gene sequence.

**Fig. 5:**
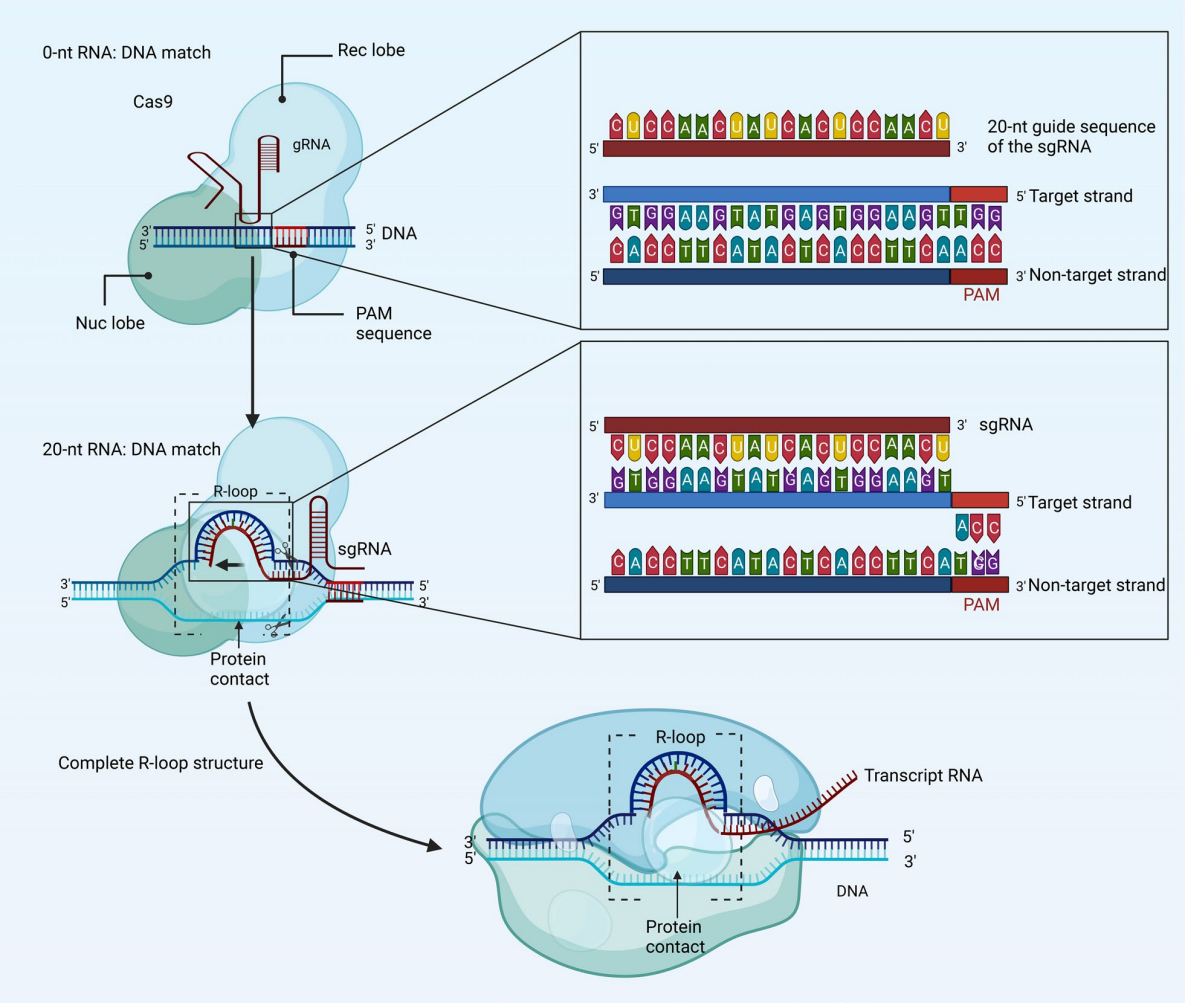
An illustration shows how Cas9 stabilizes the R-loop. In order to construct an R-loop structure, the targeted strand of DNA must link with the 20-nt of gRNA as the DNA curves. The sgRNA not matched to a target DNA sequence (0-nt RNA: DNA) that has a complementary sequence to the gRNA. DNA binding at a sequence matching the 20-nt sgRNA helps proteins accept the RNA–DNA helix and displaced non-target DNA strand. Complete R-loop formation constitutes the signal for the subsequent structure of the targeted gene. gRNA guide RNA, nt nucleotide, sgRNA single-guide RNA, Cas9 CRISPR-associated protein 9, PAM protospacer adjacent motif, Nuc nuclease, Rec recognition

The gRNA contains a short region of RNA that is complementary to the target DNA sequence, which allows the gRNA to hybridize with the DNA and form a stable duplex [115]. This RNA–DNA hybridization is specific, meaning that the gRNA will only bind to a target DNA sequence that has a complementary sequence to the gRNA (Fig. 5).

As the DNA bends, the targeted strand pairs with the 20-nt of gRNA to form an R-loop structure [116]. DNA binding at a sequence that matches the 20-nt sgRNA enhances structural changes in proteins that make accommodation for RNA–DNA helix and the displaced non-target DNA strand. Cas9 stabilizes the R-loop by interacting with both ends of the open DNA helix, creating a structural distortion of 30° in the helical bend angle [115]. After the initial melting of the DNA, the gRNA-TS gradually pairs to make a 10-base pair heteroduplex. Crystallization and X-ray analysis of a 10-nt match complex at a resolution of 2.8 Å demonstrate that the TS and non-target strand (NTS) maintain their hybrid state at the PAM-distal end of the DNA platform [112].

Variation in miRNA genes will alter the biogenesis of the miRNA and makes a significant impact on the miRNA transcription, maturation, and target specificity [117]. Thus, gene-editing approaches like the CRISPR/Cas system, which can alter genomic sequences, offer an opportunity for the management and therapy of a wide range of diseases and disorders. For example, Zhou et al. [118] showed that using CRISPR/Cas9 to target mutations in miRNA genes of rice is very effective and is one of the most dependable approaches to finding mutations. Additionally, to silence miRNA genes, Zhao et al. [119] demonstrated that Cas9 could be guided to the target strand by creating DSBs using different gRNAs. They successfully silenced miRNA genes, including those encoding carcinogenic miRNAs like miR-21 and miR-30a, by applying the CRISPR/Cas9 system.

Direct transcripts of the miRNA gene, or pri-miRNA, are subsequently converted into short mature miRNA by Drosha and Dicer enzymes [120]. CRISPR/Cas9 can cleave miRNA Drosha and Dicer processing sites, at the DNA level, which results in indels of varying sizes in the miRNA gene sequences in vivo and in vitro [121, 122]. Cells transfected with the indicated CRISPR/Cas9 constructs reduced mature miR-17, miR-200c, and miR-141 expression by almost 96% relative to control vector-transfected cells [77]. Additionally, clones with only two deletions (GT) or one insertion of nucleotides (A-T) can obstruct the exogenous expression of the miR-17 gene in addition to single clones with substantial portions of deletion (such as 6–18 bp) that prevent mature miR-17 gene synthesis.

These findings provide significant evidence for the theory that CRISPR/Cas9-induced alterations in the stem-loop structure of pri-miRNA can inhibit the synthesis of mature miRNA.

#### Cas12-based mechanism for miRNA gene targeting

Cas12 proteins, in contrast to Cas9, are large multidomain enzymes with a wide range of diversity that has led to their classification into more than ten distinct subclasses such as V-A to V-K, and other subclasses V-U [106, 123]. They vary in the processes via which they form RNPs, the RNPs themselves, and the nucleic acids to which they bind. RNPs can be functional when guided by either a single crRNA [124] or a hybrid of a crRNA and a trans-activating CRISPR RNA (tracrRNA) [125] or a crRNA and short-complementarity untranslated RNA (scoutRNA) [126]. Generally, Rec and Nuc lobes, similar to those found in Cas9, are present in even the smallest Cas12 effectors, like Cas14 (referred as Cas12f; subtype V-F) [127, 128], Cas12j (subtype V-J) [129] and Cas12g (subtype V-G) [130]. Beyond the similar bilobed structure, structural analyzes of different Cas12 proteins revealed remarkable homogeneity. Specifically, a crRNA oligonucleotide-binding domain and the RuvC domain give rise to the Rec lobe domains (Rec1 and Rec2) and the DNA-loading “nuclease” (Nuc) or zinc-ribbon domains, which together provide a highly adaptable platform. Other short domains are sometimes fused or added into this basic structure to facilitate PAM identification and NTS binding or to direct recruiting efforts via a zinc-finger motif.

Cas12a is an example of a Cas12 protein that uses a method similar to that used by other Cas12 proteins for binding and cutting DNA. Cas12a forms an adaptable “open” conformation in the absence of a gRNA, and a “closed” conformation that is prepared for PAM recognition upon crRNA binding [131, 132]. The RuvC active site for nuclease suppression is structurally occluded by the Rec domains in the closed conformation [131]. Rec domains rearrange to make space for the heteroduplex form when a dsDNA target is unwound in a PAM-dependent manner and then hybridizes with a crRNA [133]. This conformational change occurs simultaneously with the activation and opening of the RuvC active site, which successively cleaves the single-stranded NTS and then the TS to produce a 5′-overhang staggered DNA DSB [134, 135]. Recent structural analysis of Cas12i and Cas12j’s active sites found that the single strand DNA (ssDNA) substrate coupled with two magnesium cofactors provides mechanistic detail for the two-metal ion catalysis process of Cas12 proteins [136]. Nonspecific ssDNA “shredding” in trans remains active in the RuvC domain after cis DNA cutting [137]. The structural details of DNA interrogation are still unclear, although it is speculated that DNA bending is involved in the duplex opening after the PAM [138]. Data showing that DNA distortion can reveal ssDNA segments, which are then identified by Cas12, provide validity to this mechanism [139].

In particular, the PAM-interacting domain has been observed frequently close to unraveled DNA and may play a key role in this process. However, it is unclear whether these domains and other nearby components effectively participate in DNA unwinding or only connect with the unwound DNA for stability. To advance the design of Cas12-based genome editing tools, a comprehensive understanding of the DNA interrogation mechanism is needed.

### Regions of miRNAs genes targeted with CRISPR/Cas

**Fig. 6:**
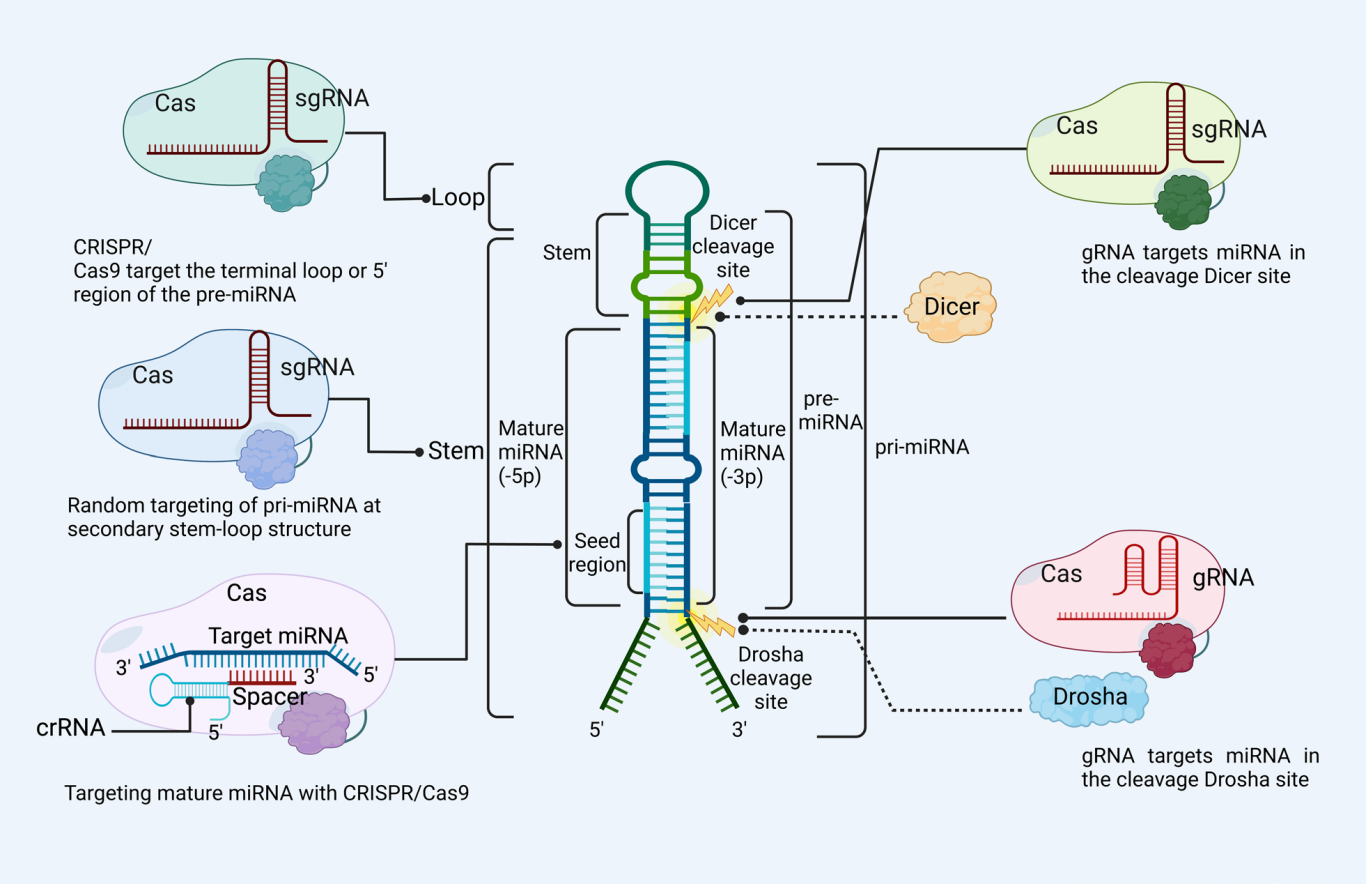
An illustration showed different targeting DNA regions of the post-transcriptional editing of miRNAs with the CRISPR/Cas system. Targeting terminal loops: CRISPR/Cas9 has the potential to inhibit the production of monoisotopic miRNAs by targeting either the terminal loop or the 5ʹ region of the pre-miRNA. Targeting secondary stem loop: Random targeting of pri-miRNA at secondary stem-loop structure by CRISPR/Cas9. Targeting mature miRNA: CRISPR/Cas9 sequences that target mature miRNA are used to successfully inhibit miRNA expression. Dicer cleavage sites: The expression levels of mature miRNAs can be successfully down-regulated by gRNA when it has been directed to target miRNA in the Dicer site. Drosha cleavage sites: gRNA successfully targets mature miRNAs at the Drosha region to bring down the expression levels of certain miRNAs. CRISPR/Cas clustered regularly interspaced short palindromic repeats/CRISPR-associated protein, gRNA guide RNA, sgRNA single-guide RNA, crRNA CRISPR RNA

Genomic engineering tools like the CRISPR/Cas system have been used to target different parts of the post-transcriptional miRNA sequences at the DNA level for therapeutic and diagnostic purposes. These parts include the Drosha cleavage site, the 5ʹ-end terminal, the upstream region of the miRNA, the secondary stem-loop structure, and/or the mature miRNA (Fig. 6). More critically, according to the Jiang et al. [140] study, which used CRISPR/Cas9 to knockout miR-93 in HeLa cells, deletion of a single nucleotide in a pre-miRNA DNA region can result in the highly specific total knockout of the targeted miRNA.

Interestingly, the CRISPR/Cas technology can be used to specifically target the terminal loop or 5ʹ region of the pre-miRNAs and inhibit their biogenesis. By using CRISPR/Cas9 to target a specific location in miR-93 in HeLa cell line, Jiang et al. [140] showed that targeting terminal loop or 5ʹ region of the pre-miRNA at the DNA level is one of the efficient ways to impair the RNA biogenesis in monoisotopic miRNA. Furthermore, they confirmed the functional knockdown by quantitative PCR analysis.

Similarly, the CRISPR/Cas technology can be used to specifically target miRNA loops and seed region of the pre-miRNAs and inhibit their biogenesis (Fig. 6). The stem-loop structure of pre-miRNAs exposes a duplex of the mature miRNA on the stem, where it can be cleaved off by the deoxyribonuclease Dicer-like 1 (DCL1) [141]. In plants [142], the pri-miRNA is turned into the miRNA duplex in the nucleus. In animals [143], this step takes place in the cytoplasm. In vitro, experiments by Chang et al. [77] showed that CRISPR/Cas9 random targeting of pri-miRNA to the secondary stem-loop structure effectively reduced miRNA families’ expression. Furthermore, they showed that the production of mature miRNA can be inhibited by introducing mutations into the stem-loop DNA structure of pri-miRNA using the CRISPR/Cas9 system.

Moreover, the CRISPR/Cas technology allows for the exact targeting of miRNA seed DNA sequences, which can then be utilized to inhibit the functions of miRNAs. The first 2–8 DNA nucleotides of a miRNA, counting from the 5ʹ end to the 3ʹ end, are known as the seed sequence, and this area has been conserved in a heptametric pattern [144]. Seed-mediated target recognition is the primary method by which miRNAs suppress translation or cause mRNA de-adenylation or disintegration [145]. Thus, the scientist tried to find out the impact of targeting the seed region of miRNA by CRISPR/Cas system to see the impact of the change on the miRNA function. For example, one of the studies performed by the Jiang et al. [140] revealed that CRISPR/Cas9 can be used as a proper tool for knocking out the miRNA-93 at the DNA level. They used HeLa cells and designed a specific gRNA to target the PAM sequence of miRNA-93 in the 5ʹ region which is one of the critical onco-miRNA in cancer prognosis [140]. They demonstrated that minor indels in the 5ʹ region of this miRNA cause sequence impairment and biogenesis inhibition, resulting in an effective and selective gene knockout.

Additionally, the CRISPR/Cas technology can be used to specifically target mature miRNAs in DNA and suppress their expression. For example, the successful silencing of miRNA expression by targeting mature miRNAs was obtained by Chung et al. [146] while aiming to shut down the expression of genes involved in drought response in rice. The team showed that targeting of mature miRNA sequences, as well as of miRNA biogenesis sites through CRISPR/Cas9 technology is a powerful tool for studying loss of function mutation in miRNAs in rice.

Further, it was demonstrated conclusively that mutations introduced by CRISPR/Cas at miRNA Drosha- and Dicer-processing sites lead to the down-regulation of mature miRNA by blocking biogenesis. CRISPR/Cas9 system can target Drosha and Dicer cleavage sites of the pri-miRNAs and inhibit their biogenesis [77]. The complex process of miRNA maturation requires the action of numerous Drosha and Dicer enzymes, which cleave particular locations in the pri-miRNA sequence to produce the complete and functional miRNA structure. The flanking and internal structures of the pri-miRNA, which determine the effectiveness of these cutting enzymes, have an impact on the specific cutting site [147]. Chang et al. [77] created a gRNA to target mature miR-17, miR-200c, and miR-141 in Dicer and Drosha cleavage sites at the DNA level because of their critical function in the biogenesis of miRNA. By transfecting cells with specific CRISPR/Cas9 constructs, they were able to reduce the production of these mature miRNAs by as much as 96% compared to cells transfected with control vectors. The results of the above study added credibility to the argument that the CRISPR/Cas9 system is highly specific for editing miRNA sequences, as it can avoid off-target effects even when modifying miRNAs within the same family or with highly conserved regions.

## Advantages of CRISPR/Cas miRNA targeting

CRISPR/Cas is a more effective, precise, and stable technique for activating or silencing miRNAs in cancer cells than recent methods. For example, the effects of an inserted mutation can be permanently integrated into the genome and passed on to the next generation of cells, which is a major advantage of using genetic engineering, especially the CRISPR/Cas system. According to Friedland et al. [148] study on *Caenorhabditis elegans*, mutations created by CRISPR/Cas9 can pass to the offspring. Likewise, according to Chang et al. [77], who cloned CRISPR/Cas9 designs with sgRNAs that target the biogenesis processing sites of certain miRNAs, CRISPR/Cas9 can substantially and specifically suppress the production of these miRNAs by up to 96%. They also demonstrated that similar results could also be obtained in vivo by transfecting mice with the CRISPR/Cas9 construct and targeting miRNA-17.

Furthermore, when compared to other methods, CRISPR/Cas offers higher accuracy and specificity (Table 3). For instance, the findings of Wu et al. [149] demonstrated that technologies based on CRISPR had great accuracy and specificity in comparison to other techniques. This precision is a result of the CRISPR/Cas system’s focus on sgRNA, which is targeted to a specific site throughout the entire genome. Moreover, Chung et al. [146] utilized CRISPR with recombinant codon-optimized Cas9 (rCas9) on 13 different types of miRNA in rice, obtaining a 59.4% rate of mutations, including mono-allelic (8.54%), bi-allelic (11.1%), and hetero-allelic combination (39.7%) mutations. Similarly, Narayanan et al. [85] proved that the CRISPR/Cas9 approach for miRNA gene knockdown can reach a high success rate in mammalian cells with minimal off-target activity. Further, they found that in mammalian cells, the CRISPR/Cas9 method for miRNA gene knockdown can achieve a success rate of 75–99% with low off-target activity. In addition, the effectiveness of miRNA silencing through CRISPR/Cas9 genome editing is also enhanced by the ability of this technique to target not only a single miRNA sequence but also several pre-miRNA structures in a single application [150].

**Table 3:** Comparison between the five most common genome editing tools

Characteristics	ZNFs	CRE-LOXP	TALENs	FLP-FRT	CRISPR
DNA binding	Targeting particular DNA sequences with an engineered protein	Targeting particular DNA sequences with Cre recombinase enzyme	Targeting particular DNA sequences with an engineered protein	Targeting particular DNA sequences with FLP recombinase enzyme	Targeting particular DNA sequences with a short RNA sequence
Sensitivity/target	Less sensitive/protein-DNA interaction	Highly sensitive/recombinase-DNA interaction	Less sensitive/protein-DNA interaction	Highly sensitive/recombinase-DNA interaction	Highly sensitive/RNA–DNA interactions
Size of recognized target	18–36 nucleotides	38 nucleotides	30–40 nucleotides	20–35 nucleotides	22 nucleotides
Ease of targeting multiple targets	Low	High	Low	High	High
Delivery	Easy	Variable (depends on the types of organism)	Difficult	Variable (depends on the types of organism)	Moderate
Design	Very complex	Simple	Complex	Simple	Simple
Nuclease-Monomer/Dimer	FokI-Dimer	Recombinase-Monomer/Dimer	FokI-Dimer	Recombinase-Monomer/Dimer	Cas/Monomer
Off-target effects	High	Low	Moderate	Lower than CRISPR	Low
Cytotoxicity	Variable to high	Variable	Low	Low	Low
Multiple targets	Difficult	Difficult	Difficult	Difficult	Easy
Cost/benefits	Expensive and time-consuming	Depends on the specific application and the resources available for genetic engineering experiments	Expensive and time-consuming	Depends on the specific application and the resources available for genetic engineering experiments	Cheap and less time needed
Mode of action	The target sequence should be surrounded by two sets of ZFN that must hybridize to each DNA strand	Cre recombinase recognizes the targeted DNA sequence and produce double strand breaks which then ligated back together in a different orientation	The target sequence must be surrounded by two sets of TALENs that must hybridize to each DNA strand	FLP recombinase recognizes the targeted DNA sequence and produce double strand breaks which then ligated back together in a different orientation	When gRNA is present, Cas may access the target DNA sequence and produce double strand breaks

*FLP* flippase, *ZNF* zinc finger proteins, *TALENs* transcription activator-like effector nucleases, *CRISPRs* clustered regularly interspaced short palindromic repeats, *FokI* flavobacterium okeanokoites I, *gRNA* guide RNA, *Cre* cyclic recombinase, *CRE-LOXP* cyclic recombinase-locus of crossing (x) over P1, *FLP-FRT* flippase-flippase recognition targets

In addition to precision and selectivity, the CRISPR/Cas system allows for the targeting of various loci within the miRNA gene. Clinical trials are continuing to explore new medicines to fight diseases in humans using a wide variety of Cas9 nuclease variants [151]. However, Cas9’s effectiveness and adaptability in genome editing are capped by its toxicity and potential for mutagenicity [152, 153]. In response to these challenges, researchers have enhanced their pursuit of CRISPR/Cas systems, which have the potential to be refined into next-generation genome editing tools. Cas12a, Cas12b, Cas12f, Cas12g, and Cas14 are some of the newest type V members in class 2 to receive scientific attention [130, 154–156]. For genome editing, Cas12a is interesting because it appears to be more precise than Cas9 [157] and has opportunities for future development [158, 159]. Cas12a’s pre-crRNA is processed into mature crRNAs due to its unique RNase activity by the proper Cas12a [90, 160], and its short crRNA (40 nt) helps overcome the size challenge in delivery via viral vectors [161]. As a result, through the delivery of numerous crRNAs on a single plasmid, this Cas12a activity has been effectively used in multiplex gene regulation to successfully edit many endogenous targets at once [162, 163].

In light of this, the CRISPR/Cas system has been widely used as a genetic engineering tool in a variety of animals, as well as in vivo and in vitro studies of human disorders, by taking advantage of the endogenous DNA repair machinery of cells.

## Challenges of targeting miRNAs by CRISPR/Cas and its strategies to overcome

### Cas9 PAM deficiency in miRNA sequence

CRISPR gRNA is composed of two main parts, crRNA and tracrRNA. The crRNA sequence targets and binds to the NGG, PAM, and the target DNA, whereas tracrRNA has a role in ensuring Cas9 nuclease activity [164]. Requiring a PAM sequence next to the target site is considered one of the main limitations of using the CRISPR/Cas9 system for targeting miRNA genes at the DNA level [165]. As miRNAs are very short sequences of nucleotides, often it is quite challenging for these small fragments to find the PAM region of the targeted miRNA. Moreover, according to the Bi et al. [166] study, most of the miRNA sequences do not contain the classic mammalian 5ʹ-NGG-3ʹ PAM region, which is essential for Cas9 protein activity. And this issue becomes even more obvious when anyone tries to target mature miRNA which is very short, usually around 20–24 nucleotides long.

To overcome this issue, advanced bioinformatics platforms can be used to assess the presence or lack of an appropriate PAM region in the target miRNA sequence. Zhou et al. [118] found that out of the 592 rice miRNAs assessed through the miRBase portal (http://www.mirbase.org/), 556 miRNAs (93.92%) presented a suitable PAM site for Cas9, showing the importance of these bioinformatics tools in successful planning experiments.

**Fig. 7:**
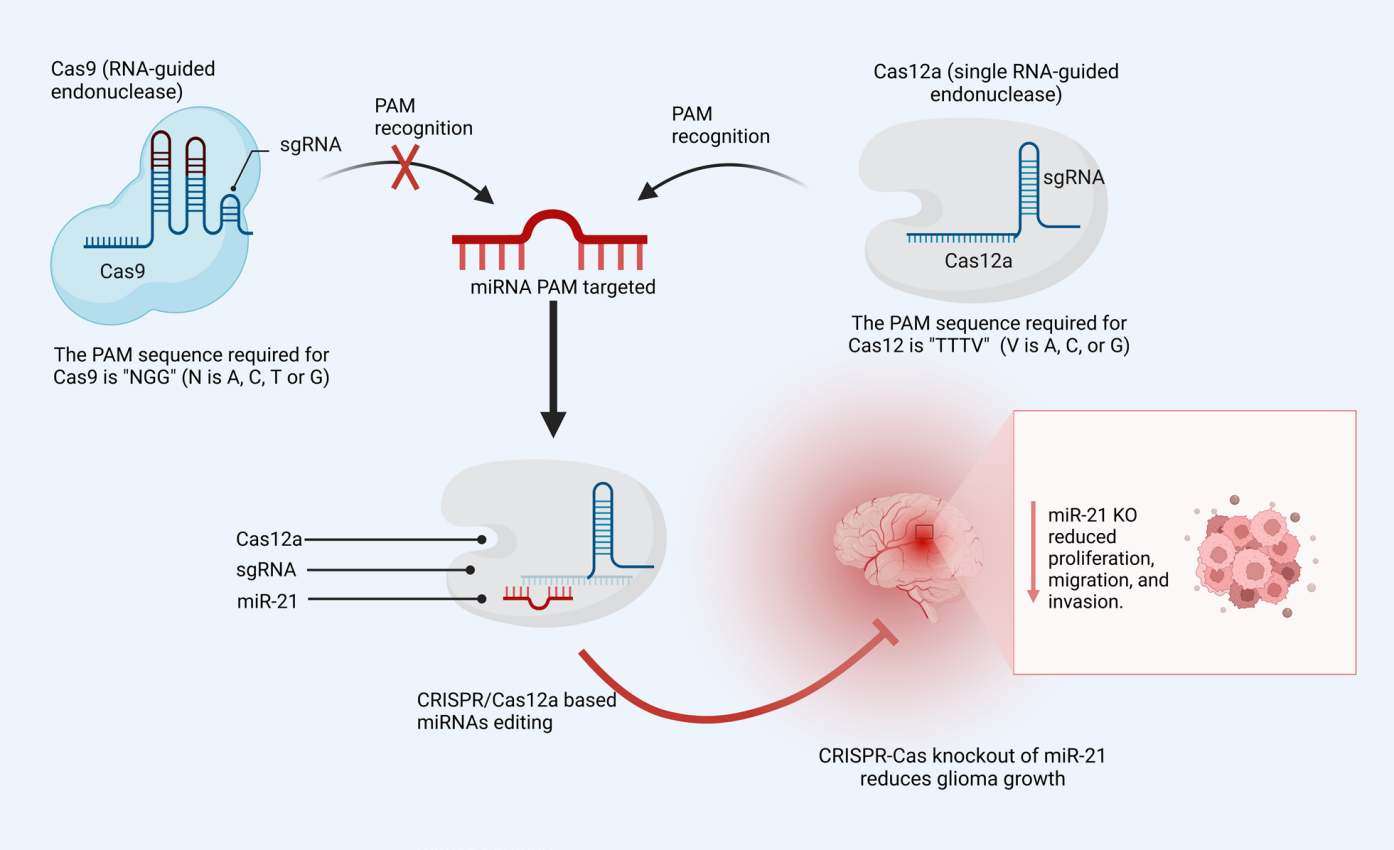
An illustration shows applying Ca12a instead of Cas9 protein, which has the ability to recognize PAM sequences in miRNA editing. By altering the miR-21 coding sequences in glioma cells, CRISPR/Cas12a decreases miR-21 expression through microenvironment cells, which controls both in vitro and in vivo cell proliferation. CRISPR clustered regularly interspaced short palindromic repeats, PAM protospacer adjacent motif, sgRNA single-guide RNA

Further, additional approaches to get around this limitation include using different Cas proteins, including Cas12a, which potentially overcome this challenge. Cas12a is a single RNA-guided endonuclease, which means it processes its own gRNAs and only needs crRNA for targeting [167]. For instance, miR-21 can reprogram microglial cells and establish a favorable environment for cancer development in glioma cells [168]. It affects native brain cell types such as endothelial cells, neurons, as well as invading monocytes and macrophages which make up-regulation of cytokine productions [169]. As a result of its ability to recognize the PAM sequences in miR-21, CRISPR/Cas12a can reduce miR-21 expression by altering the coding sequences and regulating cell proliferation in vivo and in vitro [170] (Fig. 7).

Besides that, the PAM sequence required for Cas12 is "TTTV" (where V is A, C, or G), but the PAM sequence needed for Cas9 is "NGG" (where N is A, C, T, or G) [105]. Nevertheless, scientists have found a unique property of Cas12 that can be used for something other than editing the genome such as cutting ssDNA. These recently uncovered features of Cas12 make CRISPR an attractive new area for targeting miRNAs in cancer therapy.

Furthermore, multiple Cas proteins, as shown in Table 4 [107, 141, 154, 171–179], bind to various PAM sites. Therefore, the success rate of CRISPR-based miRNA editing could be improved by using a variety of Cas proteins as well.

**Table 4:** List of Cas proteins with their PAM sequence targets

CRISPR/Cas system	Types of Cas	Organism isolated from	Major application area	PAM sequence	PAM location relative to target base	Cas size (in amino acids)	References
Type II CRISPR/Cas system	SpCas9	*Streptococcus pyogenes*	Gene editing	NGG	Downstream of the target site	1368 AA	[171]
	xCas9	Modified from SpCas9	Gene editing	NG, GAA and GAT	Downstream of the target site	1368 AA	[107]
	SaCas9	*Staphylococcus aureus*	Gene editing	NGRRT or NGRRN	Downstream of the target site	1058 AA	[172]
	SaCas9-KKH	*Staphylococcus aureus* Cas9	Gene editing	NNNRRT	Downstream of the target site	1058 AA	[173]
	StCas9	*Streptococcus thermophilus*	Gene editing	NNAGAAW	DSBs	1121 AA	[174]
	ScCas9	*Streptococcus canis*	Gene editing	NNG	Downstream of the target site	1375 AA	[175]
	CjCas9	*Campylobacter jejuni*	Efficient genome editing	NNNVRYM	Downstream of the target site	984 AA	[176]
Type V CRISPR/Cas system	LbCas12a	*Lachnospiraceae bacterium*	Gene editing and diagnosis	TTTV	Upstream of the target site	1228 AA	[177]
	enAsCas12a	*Streptococcus pyogenes*	Gene editing and diagnosis	TTTV	Upstream of the target site	–	[178]
	Cas12f	*Selenomonas sputigena*	Diagnosis	TTTV	Double strand cleavage	422–603 AA	[154]
Type V CRISPR/Cas system	Cas14b	*Extremophile archaea*	Gene editing and diagnosis	T-rich PAM sequences, e.g., TTTA for dsDNA cleavage, no PAM sequence requirement for ssDNA	Upstream of the target site	400–700 AA	[141]
Type I CRISPR/Cas system	Cas3	*Escherichia coli*	Diagnosis	No PAM sequence requirement	Upstream of the target site	1224	[179]

*AA* amino acids, *DSBs* double strand break, *PAM* protospacer adjacent motif, *SpCas9* Streptococcus pyogenes Cas9, *SaCas9* Staphylococcus aureus Cas9, *StCas9* Streptococcus thermophilus, *ScCas9* Streptococcus canis Cas9, *LbCas12a* Lachnospiraceae bacterium Cas12a, *CjCas9* Campylobacter jejuni Cas9, *CRISPR/Cas* clustered regularly interspaced short palindromic repeats/CRISPR-associated protein, *T* thymine, *A* adenine, *G* guanine, *V* adenine, cytosine, or guanine, *N* any nucleotide, *R* purine

### Short sequence of miRNA

One drawback of CRISPR gene editing is the challenge of detecting sgRNAs besides PAM, particularly when looking at miRNAs, which have a short nucleotide sequence of 20 to 24 bases. The same issue is also present when using other inhibitors, such as antisense RNAs, to target miRNA. Moreover, not all sgRNA is efficient and working properly. Thus, sometimes a PAM sequence is available in a miRNA gene, the sgRNA might not be efficient enough to knock down the miRNA.

To design a reliable and effective sgRNA, it is important to use properly in silico techniques to show all the predictions [180]. Another way to overcome this issue is to use more than one Cas protein to target different parts of the interested gene. For instance, Godden et al. [87] showed that when two sgRNAs are used together, the targeted miRNA gene loses all of its functions in embryos. Additionally, the discovery of new and more Cas nucleases with broader PAM recognition sequences gives more opportunities to design gRNAs in different parts of miRNA sequences [181].

### Off-target effects in CRISPR/Cas-mediated miRNA editing

Off-targeting, which results from a gRNA mismatch binding to the wrong target, is one of the key challenges of using the CRISPR/Cas system [182]. All gene silencing approaches, including the CRISPR/Cas system, is characterized by off-target effects, which have high frequency [183]. Basically, off-targeting happens when the gRNA binds to the wrong target due to similarities between different sequences of the same genome, especially in mammals, and it might lead to further mutation and disorders [5].

CRISPR has attracted a lot of attention as a potential new gene editing tool over the past two decades. However, it has proven of limited use due to its tendency to be off-target. Because of this, a growing number of studies have spent the last few years working to enhance the system’s editing abilities while also reducing the number of undesired consequences. Include in particular the following strategies to overcome off-targeting:

Firstly, increasing sgRNA specificity. The specificity of sgRNA and target DNA recognition is determined by the number of base pairs in the region of the sgRNA, typically 10–12 bp [149]. The off-target effects are also modified by the remaining sequence to various levels. The efficacy of sgRNA-based gene editing was found to increase in direct correlation with the GC level of the seed region. The off-target effect is reduced or disappears when there are three or more base mismatches between the sgRNA seed region sequence and the DNA sequence at the off-target position [184]. Accordingly, 40–60% of the GC content can be used during sgRNA sequence design. The specificity of sgRNA can be increased by using sequences that are highly dissimilar to those of the off-target genes [184].

In addition, the specificity of a sgRNA is highly linked to its length. Using shorter sgRNA sequences (those with less than 20-nt) has been shown to decrease the off-target effects without reducing the efficiency of gene editing [185]. Shortening sgRNA sequences, however, may not improve specificity, and may even decrease gene editing efficiency. Therefore, more studies are needed to confirm the effectiveness of the method of decreasing the off-target effect by decreasing sgRNA length. Furthermore, adding two guanines (called ggX20 sgRNAs) to the 5ʹ end of a sgRNA in place of the matching GX19 sgRNAs during the design process is another simple alteration that may be made to boost the specificity of the sgRNA and decrease its off-target effect [183, 186]. Off-targeting can also be reduced by using sequence gRNA design and off-target evaluation sites that are available online (Table 5 [187–201]).

**Table 5:** List of main Bioinformatics tools with their features and types of Cas proteins

Tool name	Organism	Cas nuclease enzyme	Characteristics	Website	References
CRISPResso2	Human and mouse	Cas9 Cpf1	Amplicon sequencing and its interpretation for genome editing	http://crispresso2.pinellolab.org/submission	[187]
Cas-Analyzer (CRISPR-GA)	Human, mouse, rat, drosophila, zebrafish	NmCas9, SpCas9, SaCas9, StCas9, AsCpf1/LbCpf1	Editing genomes and creating synthetic nucleases (programmable nucleases)	https://crispr-ga.net/	[188]
CRISPOR	Human, Arthropoda	Cas9 orthologues, Cas variants	Primer sets, genomic location of targets, and numerous predictive models	http://crispor.tefor.net/	[189]
TIDE/TIDER	–	SpCas9, SaCas9, St1Cas9, NmCas9, AsCpf1, FnCpf1, LbCpf1	Large-scale induced mutations in the editing site are identified with a custom deconstruction approach	http://shinyapps.datacurators.nl/tider/	[190]
CHOPCHOP	Human, mouse, zebrafish, bacteria	Cas9, Cas12, Cas13, TALEN	Providing multiple predictive models; visualizing genomic location of targets and genes; and providing primers	https://chopchop.cbu.uib.no/	[191]
CRISPR RGEN Tools	Human	Cas9	Microhomology-predictor predicts out-of-frame scores, predicts probable off-target numbers, and can be used independently	http://www.rgenome.net/	[192]
E-CRISP	Human	SpCas9	Genome-scale library construction in a practical manner; freely available and regularly updated	http://www.e-crisp.org/E-CRISP/	[193]
CRISPRscan	Human	Cas9, Cas12	Coding-gene sgRNA design and genome browser tracks	https://www.crisprscan.org/	[194]
CCTop	Human, mouse model	Cas9	Easy to use, highly customizable, single and multiple query searching; identifying mismatches; estimating off-target impacts; sgRNA efficiency prediction	https://cctop.cos.uni-heidelberg.de:8043/	[195]
CRISTA	–	SpCas9	Offering a machine learning framework for identifying false positives and prioritizing targets, which is more precise than existing methods	https://crista.tau.ac.il/	[196]
DeepCRISPR	Human	SpCas9	Predicting off-target effects by factoring in epigenetic data	https://github.com/bm2-lab/DeepCRISPR	[197]
WU-CRISPR	Human, mouse	SpCas9	Offering an ML algorithm that has been highest prediction scores	https://bio.tools/wu-crispr	[198]
CRISPRz	Zebrafish, human, mouse	SpCas9	Specific for a wide range of species and cell lines	https://research.nhgri.nih.gov/CRISPRz/?mode=search	[199]
AsCRISPR	Human, mouse	SpCas9, AaCas12b, AsCpf1, CasX	Creating sgRNAs for genomic sites with specified alleles	https://bio.tools/AsCRISPR	[200]
CRISPRlnc	Human	SpCas9	Making a validated sgRNA database for lncRNAs downloadable	http://www.crisprlnc.org/	[201]

*TALENs* transcription activator-like effector nucleases, *sgRNA* single-guide RNA, *lncRNAs* long non-coding RNA, *ML* machine learning, *NmCas9* Neisseria meningitidis cas9, *SpCas9* Streptococcus pyogenes Cas9, *SaCas9* Staphylococcus aureus Cas9, *StCas9* Streptococcus thermophilus Cas9, *AsCpf1/LbCpf1* Acidaminococcus Cpf1/Lachnospiraceae Cpf1, *FnCpf1* Novicida U112, *AaCas12b* Alicyclobacillus acidiphilus, *CRISPR* clustered regularly interspaced short palindromic repeats, *CRISTA* CRISPR target assessment, *TIDER* threat Intelligence deficiency report

Secondly, Cas-sgRNA dosage control. One of the other strategies that can be used to minimize the consequences of off-target effects is controlling the amount of sgRNA or Cas protein produced. For example, when Pattanayak et al. [202] cut missed loci in the HEK293T cells’ genomes, they discovered that short sequences, low sgRNA activity compared to sequence length, high activity has better specificity, and high amounts of sgRNA-Cas9 compounds can cut close internal sites or PAM sequences. Cas9, which has DSB activity, is continuously expressed in cells, which could enhance the risk of off-target sequences. This risk can be minimized by blocking antibodies or inhibitors of the Cas9 protein, which limits the off-target effect. Regulating the concentration of sgRNA and Cas9 nucleases can lower the off-target risk, but this will also reduce the corresponding genome editing ability. Thus, it is necessary to balance the efficiency of gene editing against the possibility of producing an off-target effect. Recent studies proved that decreasing the Cas9:gRNA complex ratio to 1:2 or 1:3 improves knockout efficacy and successfully decreases off-target effects [108, 203].

The third strategy is the chemical modification of sgRNA. Besides the current strategies, chemical modification of sgRNA is also an effective way to minimize off-target effects. Chemical modifications to crRNA include 2-fluoro ribose, and 2-O-methyl-3ʹ-thiophosphate (MS), which can strengthen the selectivity of Cas9 endonuclease and the stability of sgRNA [204, 205]. A further factor crucial to successful gene manipulation is the strategy for optimizing off-target effect identification. Off-target effects of CRISPR/Cas9 can be detected using tools like integration-deficient lentiviral vectors (IDLVs) containing integrase defects, and this method can identify an off-target frequency of at least 1% [206].

### Targeting multi-sites of gene by one miRNA or targeting one site/pathway by multiple miRNAs

As discussed in the previous section, miRNAs undergo a complex molecular pathway within the cell, which is not fully elucidated. According to “seed sequence matching” bioinformatics research, a single miRNA can control hundreds of target genes, while many miRNAs can trigger a single gene. For example, the miR-17-92 cluster suppresses cyclin-dependent kinase inhibitor 1A (CDKN1A), E2F transcription factor 1 (E2F1), and PTEN, which can cause up-regulated cell growth; miR-200 targets ZEBs to induce E-cadherin, which inhibits EMT [77]. Therefore, there is a potential for different consequences when researchers attempt to target a single miRNA to reduce the amount of certain proteins by focusing on the miRNA that complements its mRNA.

Conversely, the protein-synthesis pathway of a gene can be regulated by more than one miRNA [207]. For example, using a high-throughput luciferase reporter screen, Wu et al. [208] found that 28 miRNAs can directly reduce p21Cip1/Waf1 or CDKN1A by targeting its 3ʹ untranslated region. Furthermore, many of these miRNAs were found to be elevated in malignancies, suggesting that they could act as oncogenesis modulators.

**Fig. 8:**
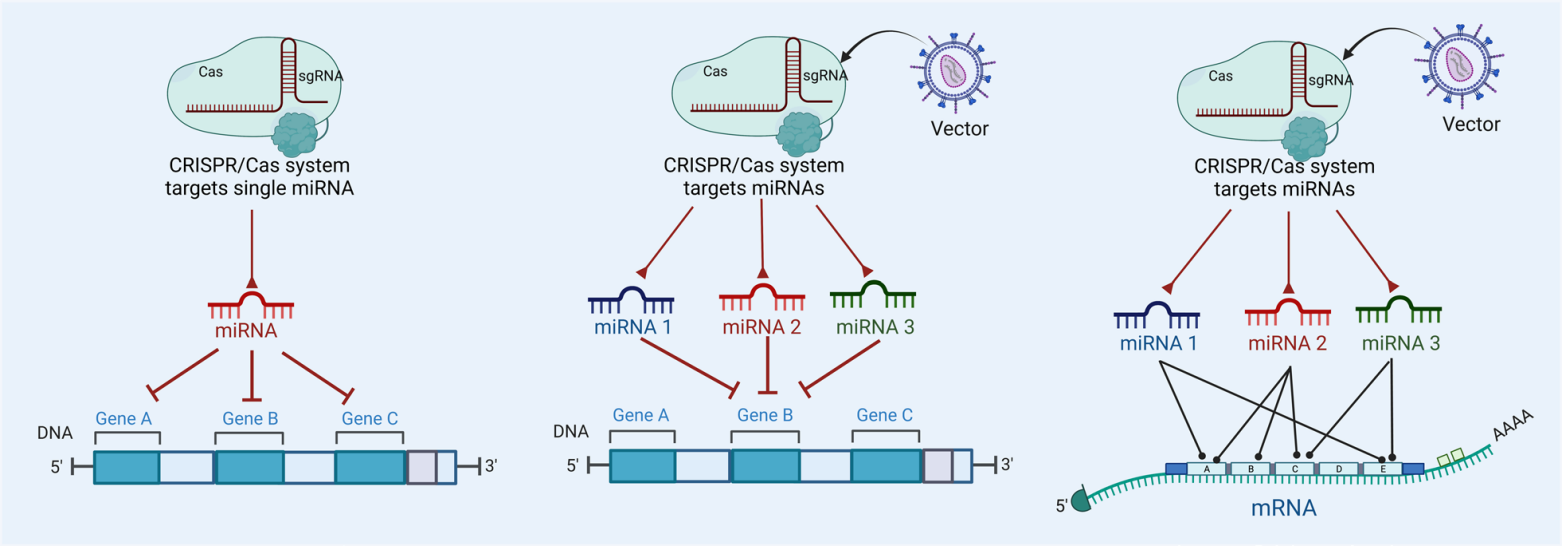
An illustration shows the main strategy to overcome multiple sites targeting or one site targeting through the CRISPR/Cas system. The CRISPR/Cas system targets one miRNA, which in turn suppresses miRNA to bind numerous genes and limit the synthesis of tumor protein. The CRISPR/Cas system is designed to target numerous miRNAs, which in turn restrict the same oncogenic gene, thereby limiting the growth of tumors. The CRISPR/Cas system is intended to target a large number of miRNAs, which then block the activity of the same oncogenic gene. As a result, the progression of tumor cell is suppressed or slowed down. CRISPR clustered regularly interspaced short palindromic repeats, sgRNA single-guide RNA, Cas CRISPR-associated protein

To obtain a complete therapeutic effect, targeting just one miRNA region in the genome is not enough. As shown in Fig. 8, one miRNA or more than one miRNA can target the same gene, which means that targeting just one miRNA is not enough to interrupt a disease as long as more than one miRNA controls the same gene. Therefore, to overcome this challenge, it is best to create or develop multiple gRNA that can be utilized to target different genomic loci or mature miRNA genomic sequences (Fig. 8). Kabadi et al. [209] previously developed a single lentiviral strategy to express and deliver a Cas9 nuclease and three to four sgRNAs transcribed from separate and distinct RNA polymerase III promoters. Interestingly, multiple gene editing and prolonged transcriptional activation were facilitated by the high levels of expression of individual sgRNAs in HEK293T and primary human dermal fibroblasts cells. This indicates the potential utility of this approach in miRNA-based biomedicine [209]. Moreover, there are different vectors with different capacities, such as adenovirus vectors, which can successfully express eight different multiplex gRNAs [210]. This strategy holds promise for minimizing the number of CRISPR/Cas components used and the risk of undesirable side effects of the co-delivery of several sgRNAs encoding constructs.

### Delivery challenges of CRISPR/Cas system both in vivo and in vitro

Delivery of CRISPR/Cas presents numerous challenges, such as the selection of a good, safe, and precise vector. Inappropriate vectors are associated with a higher risk of toxicity and off-targeting [5]. Likewise, CRISPR/Cas delivery is far more challenging to get precisely in vivo than in vitro [211]. Toxicity, size capacity, and mismatching are the three main types of in vivo delivery difficulties presented by the CRISPR/Cas system [5, 9, 212, 213].

CRISPR/Cas vectors can be broadly classified into two types: viral and non-viral [214]. Interestingly, an increasing number of studies are selecting lentivirus as their vector of choice. However, using a viral vector in vivo has a number of drawbacks, including insertional restriction, immune response, and size capacity (Table 6 [78, 87, 88, 119, 215–222]). Moreover, the risks of off-targeting and further mutation rise with prolonged expansion following insertion [223].

**Table 6:** Summarizes the most common delivery approaches that can be used for both in vivo and in vitro CRISPR/Cas system delivery

Delivery methods	Carrying capacity	Toxicity	Biosafety level	Advantages	Challenges	Strategies	References
Adenovirus	38 kb	High	BSL-2	The host genome disruption risk is low Having trouble transducing certain cell types	High immunogenicity	Targeting immune privilege orangs	[215, 216]
AAV in vitro	4.7 kb	Very low	BSL-1	Animal models with long-term transgenic expression AAV has not been linked to any diseases in humans Very low immunogenicity	Limited size capacity Preexisting immunity to natural serotypes Exposes constantly for a long time after injection Hepatotoxicity	Splicing the Cas protein into two vector Targeting infant and immune privilege organs Using anti-Cas proteins	[88]
Retrovirus	8 kb	Low	BSL-2+	Ability to transform their single-stranded RNA genome into a double stranded DNA molecule Ability to stably integrate into the target cell genome	–	–	[119]
Lentiviral vector	8 kb	Moderate	BSL-2+	Large genetic capacity Ability to transduce both dividing and non-dividing cells	De novo protein expression may cause immunological responses that result in the removal of transduced cells and the production of antibodies that block the action of released factors	Tacrolimus, cyclophosphamide, and cyclosporine can stop the production and release of cytokines as well as the activation and expansion of T cells	[78]
Baculovirus	38 kb	Very low	BSL-1	Flexible enough to contain many genes or big inserts By infecting insect cells, recombinant baculoviruses can easily be created and yield high titers	–	–	[217, 218]
Electroporation	15 kb	Very low	BSL-2	Takes less time and cost Used in in vivo, in vitro, and ex vivo research	Limited experiences in vivo	–	[219, 220]
Microinjection	No size limitation	–	BSL-1	Successful approach to inject macromolecules into embryos Guaranty of delivery to the targeted cell	Time consuming Require skill and facilities Performed generally in vitro	A high level of sophistication and physical skills are needed to reduce cell damage	[87, 221]
Inorganic compound-based nanoparticle	–	Very low	BSL-1	Non immunogenic Low cytotoxicity High packaging capacity	Delivery efficiency is low	–	[222]
Polymeric delivery system	–	Very low	BSL-1	Non immunogenic Transient expression High packaging capacity	Cytotoxicity In vivo efficacy is low	–	[222]

*AAV* adeno-associated virus, *Cas* CRISPR-associated protein

## Strategy to overcome delivery challenges

Vector capacity is a significant limitation during CRISPR/Cas delivery, especially when viral vectors such as adeno-associated virus (AAV) are used. However, several strategies can be used to overcome or minimize this problem, like picking up a smaller Cas protein or using two vectors instead of one. Several Cas proteins have been categorized or selected according to their molecular weight; for example, a smaller version of Cas9 was discovered in *Staphylococcus aureus*, showing that size optimizations have been performed. This Cas9 variant is 1 kb smaller than the original Cas9 from *Streptococcus pyogenes*; therefore, it may be integrated into a single AAV vector [224]. Furthermore, Cas14 has a two-fold smaller molecular size than Cas9 [225]. Secondly, more than one viral vector can be used to deliver the CRISPR/Cas system [9, 226]. For example, using two vectors rather than one reduces the off-targeting risk, which rises in parallel with the vector’s size [5, 224].

On the other hand, non-viral vectors are delivery systems that can be used to transport therapeutic molecules, such as CRISPR/Cas systems, into target cells without the use of viral vectors in both in vivo and in vitro [223]. The advantages of using non-viral vectors for CRISPR/Cas delivery into cancer cells include their safety, low immunogenicity, ease of preparation, versatility, minimal off-targeting, and less exposure to nuclease [223, 227, 228]. However, non-viral vectors have some crucial drawbacks such as degradation in vivo experiments, varied biocompatibility and toxicity, low delivery efficiency, and restricted delivery efficiency [228].

Several types of non-viral vectors can be used for CRISPR/Cas delivery, including liposomes, polymeric nanoparticles, and viral-like particles. Liposomes are spherical vesicles composed of a lipid bilayer that can be used to encapsulate nucleic acids, including CRISPR/Cas components [229]. Polymeric nanoparticles are composed of synthetic polymers that can also be used to encapsulate CRISPR/Cas components [230]. Finally, viral-like particles are self-assembling protein cages that can be used to deliver CRISPR/Cas components [231].

## Conclusions and perspectives

The current study underlines the background of the CRISPR/Cas system in miRNA-based cancer therapy. The CRISPR/Cas system provides new insights into cancer therapeutics that were previously unexplored in our understanding of the non-coding genome. With the development of CRISPR/Cas-based gene editing technology, it is now possible to target mutations in a precise and permanent way. Short non-coding RNAs like miRNA can also be targeted in a precise way at the DNA level. Therapeutic genome editing based on CRISPR/Cas-miRNA targeting is moving from preliminary research to preclinical development. The challenging task of identifying miRNA targets has been approached in a number of ways, including the application of CRISPR screening and miRNA gene alteration. When there is a miRNA mutant, CRISPR knockout libraries can be used to find target genes whose mutation fixes the miRNA mutant phenotype. Interestingly, the biological role of specific sites can be explored through specific sgRNA libraries that target miRNA binding sites. Custom libraries can be delivered into wild-type cells to select cells with binding site mutations that mimic the oncogenic miRNA. The CRISPR/Cas technology is currently undergoing clinical trials for the treatment of cancer, and its application in cancer immunotherapy and the inactivation of cancer-causing viral infections holds promise for addressing altered cancer cells and extending the scope of cancer therapeutic targets based on miRNA therapy. Currently, the potential use of CRISPR/Cas as a miRNA targeting platform in cancer therapy has only been partially explored, and it needs further studies.

## Data Availability

The datasets generated and analyzed during the current study are available from the corresponding author on reasonable request.

## Abbreviations

AAV: Adeno-associated virus
ADAR: Adenosine deaminase RNA specific
AGO: Argonaute
CDKN1A: Cyclin-dependent kinase inhibitor 1A
CRISPR: Clustered regulatory interspaced short palindromic repeats
crRNA-TS: CRISPR RNA-targeted sequence
CYP: Cytochrome P450
CYP7A1: Cholesterol 7 alpha-hydroxylase
DCL1: Dicer-like 1
DSBs: Double strand breaks
E2F1: E2F transcription factor 1
EGFR: Epidermal growth factor receptor
EMT: Epithelial-to-mesenchymal transition
gRNA: Guide RNA
HDR: Homology-directed repair
IDLVs: Integration-deficient lentiviral vectors
NF-κB: Nuclear factor-κB
NHEJ: Non-homologous end joining
NTS: Non-target strand
Nuc lobe: Nuclease lobe
ORF: Open reading frame
PAM: Protospacer adjacent motif
PIK3R2: Phosphatidylinositol 3-kinase regulatory subunit 2
Pol II: Polymerase II
pre-crRNAs: Precursor CRISPR-RNA
pri-miRNA: Primary miRNA
PTEN: Phosphatase and tensin homolog
Rec lobe: Recognition lobe
RISC: RNA-induced silencing complex
RNP: Ribonucleoprotein
scoutRNA: Short-complementarity untranslated RNA
sgRNA: Single-guide RNA
SOCS1: Suppressor of cytokine signaling 1
ssDNA: Single strand DNA
SSNs: Sequence specific nucleases
TALEN: Transcription activator-like effector nucleases
TracrRNA: Trans-activating CRISPR RNA
TRBP: Tar RNA-binding protein
TS: Target strand
UTR: Untranslated region
VEGF-A: Vascular endothelial growth factor A
XPO5: Exportin 5
ZEB1: Zinc finger E-box-binding homeobox 1
ZEB2: Zinc Finger E-box binding homeobox 2
ZEBs: Zinc finger E-box binding homeoboxs
ZFN: Zinc finger nuclease
